# Effect of Propolis on the Quality Characteristics and Shelf Life of Raw Beef Meatballs during Refrigerated Storage

**DOI:** 10.17113/ftb.63.03.25.8774

**Published:** 2025-09-30

**Authors:** Aslihan Cevik Ozkir, Ahmet Sukru Demirci, Harun Uran, Recep Gunes, Berkay Kopuk, Bayram Cetin

**Affiliations:** 1 Tekirdağ Namık Kemal University, Faculty of Agriculture, Dept. of Food Engineering, 59030, Tekirdağ, Türkiye; 2 Kırklareli University, Faculty of Engineering, Dept. of Food Engineering, Kayalı Campus, 39000, Kırklareli, Türkiye; 3 Kırklareli University, Food R&D, Innovation and Entrepreneurship Application and Research Center, Kayalı Campus, 39000, Kırklareli, Türkiye

**Keywords:** meatballs, propolis, meat quality, shelf life

## Abstract

**Research background:**

Recently, natural ingredients have come to the fore instead of synthetic additives in meat and meat products. In this context, the use of propolis extracts, a natural bee product, prepared with different solvents is quite widespread. From this perspective, this study investigates the contribution of ethanolic extract of propolis on the shelf life of beef meatballs and the extent to which it maintains the quality characteristics of the samples during storage at 4 °C.

**Experimental approach:**

In this study, an ethanolic extract of 0.1, 0.3, 0.5 and 1 % of propolis was added to meatball samples. After preparation, the samples were packaged and stored at 4 °C. During storage, the meatball samples were subjected to physicochemical, microbiological and sensory analysis.

**Results and conclusions:**

The addition of propolis did not affect significantly the water activity values. However, significantly lower pH values were observed during prolonged storage, especially in the samples containing higher amounts of propolis. The addition of propolis also effectively delayed oxidation and there was an amount-dependent decrease in TBARS values. The use of propolis in the preparation of meatballs did not have a significant effect on the initial CIELab parameters of the samples, but changes in a* and b* values ​​were observed at the end of storage compared to the control sample. A significant increase in the total phenolic content as well as the DPPH˙ and ABTS^+^ radical scavenging activities of the meatballs was observed depending on the propolis amount. Considering the results of microbiological analysis, it was found that propolis could increase the microbiological quality of the meatballs, but the addition of more than 0.5 % propolis affected the overall acceptability of the samples.

**Novelty and scientific contribution:**

As a result, the addition of certain amounts of propolis was found to be a potential alternative to synthetic counterparts that could be used to preserve refrigerated meatballs to delay oxidation and microbial spoilage without affecting the sensory properties of the samples.

## INTRODUCTION

It is well-known that meat is an important source of protein and other nutrients (*1*). However, meat products are susceptible to spoilage due to their composition. Oxidative changes and microbial growth are therefore the two important factors that determine the storage stability of these products. In meat products such as minced meat, oxidative stability depends on the interaction between endogenous pro- and antioxidant substances (*2*, *3*). For instance, lipid and protein oxidations reduce the quality of meat and meat products, which ultimately affects consumer acceptance. Therefore, artificial antioxidant compounds such as butylated hydroxyanisole (BHA), butylated hydroxytoluene (BHT), propyl gallate (PG), *tert*-butylhydroquinone (TBHQ) and erythorbates are widely used to reduce or inhibit the oxidative deterioration of these products (*4*, *5*).

Another important factor for the quality and safety of meat products is microbial activity. In general, fresh meat and meat products have a water activity (*a*_w_) value higher than 0.85 and a pH that favours spoilage and pathogenic microorganisms to survive in the product (*6*). For this reason, synthetic antimicrobials such as chlorides, nitrites, sulfites and sorbates are widely used to extend refrigerated storage as the most common method for the preservation of meat products (*7*). However, the use of these synthetic antimicrobials and antioxidants in food products has been associated with carcinogenic effects and health risks. To address these concerns, it may be necessary and useful to develop and apply natural ingredients with both antioxidant and antibacterial activity that ensure safe consumption of various meat products (*8*-*11*).

Moreover, consumer demand is increasing for more natural and minimally processed products with better bioactive properties and sensory quality. In this context, some processing strategies including the use of non-thermal processing methods, the addition of polyphenols from vegetable and fruit by-products, and the incorporation of different herbs and spices, are being investigated to improve the oxidative and microbial quality of meat products and thus extend the shelf life of the samples (*11*). In this respect, a resinous bee product called propolis, which has significant antibacterial and antioxidant properties, stands out as a potential natural ingredient for the replacement of synthetic additives in meat products (*5*, *12*, *13*). Propolis is known to be a hive product produced by honey bees (*Apis mellifera*) by collecting resins from plant flowers and exudates and combining them with salivary secretions and beeswax. The bioactive properties of propolis can vary depending on factors such as botanical origin, chemical composition, season, age bees and area or time of collection. Propolis consists of more than 300 different compounds, mainly plant resins (50 %), beeswax (30 %), essential oils (10 %), pollen (10 %) and vitamins (B_1_, B_2_, B_6_, C and E), benzoic acid and derivatives, flavonoids and derivatives, amino acids and minerals (*14*-*17*). The antioxidant (*18*), antibacterial (*19*), antifungal (*20*), anti-inflammatory (*21*) and anticarcinogenic (*22*) properties of propolis, combined with the fact that the substances found in propolis are commonly present in food, make it an excellent candidate as an alternative to conventional synthetic preservatives in meat and meat products.

Nevertheless, the use of propolis in food preservation is limited because it can affect the sensory properties of the product due to its intense taste and odour. Therefore, it would be very important to determine the amount of propolis that can be used for preservation purposes without changing the sensory properties of the products. For instance, adding approx. 0.5 % propolis extract to various food products, such as fish sausages and poultry products, provides good sensory results (*17*). However, the literature about the effect of propolis on different quality factors and sensory properties of meat and meat products is very limited. Therefore, the aim of this study is to investigate the effects of different amounts of propolis on the quality properties of meatballs and to determine the acceptable amount that can ensure preservation during refrigerated storage (9 days at 4 °C).

## MATERIALS AND METHODS

### Materials

The beef containing 20 % fat, all seasonings (spices) and other ingredients used to make meatball samples were purchased from a local producer in Kırklareli, Türkiye. Ethanolic extract of propolis (30 %, *m*/*V*) was supplied by Tekirdağ Namık Kemal University, Faculty of Agriculture, Food Engineering Department, Tekirdağ, Türkiye. The extract was prepared using 70 % ethanol (Merck KGaA, Darmstadt, Germany) and a crude propolis sample taken from an apiary in the Mersin region, located in the southern part of Türkiye. Then, the sample was homogenized (T 25 ULTRA-TURRAX®; IKA, Staufen, Germany) for 30 min and kept at room temperature in the dark for 15 days. Finally, the prepared extract was filtered through Whatman No. 4 filter paper (Millipore, Burlington, MA, USA) and stored at 4 °C until further analysis (*23*). All chemicals used for analysis were of analytical grade and supplied by Merck KGaA.

### Preparation of meatballs

The beef used for the preparation of meatballs was first cleaned from cartilage and nerves. Then, the lean beef (84 %), bread crumbs (10 %) and red onion (3 %) were ground in a meat grinder (Arı Makina, Istanbul, Türkiye). After that, 0.5 % cumin, 0.5 % black pepper powder and 2 % table salt were added to the dough and kneaded by hand using sterile gloves for 30 min to obtain a homogeneous mixture (*24*). The obtained meat dough was divided into five batches and propolis was added at different amounts: control (no propolis added), M0.1, M0.3, M0.5 and M1 (meatballs containing 0.1, 0.3, 0.5 and 1 % propolis, respectively). Raw meatball samples (30 g, 5 cm diameter) were shaped by hand, transferred into disposable food grade plastic (PET) containers with lids and then tightly sealed, labelled and stored aerobically in a refrigerator at 4 °C throughout the study (9 days).

### Physicochemical analysis

Meatball samples (10 g) were homogenized (Hg-15D; Daihan, Seoul, Korea) in 90 mL of distilled water for 1 min and the pH was determined by immersing the probe of the pH meter (HI 2211; Hanna Instruments, Smithfield, RI, USA). Before determining water activity (*a*_w_), the samples were allowed to equilibrate at 25 °C for 30 min and the *a*_w_ was measured using a benchtop *a*_w_ meter (LabSwift; Novasina, Lachen, Switzerland). To determine lipid oxidation in raw meatballs, thiobarbituric acid reactive substance (TBARS) was used according to the method proposed by Gokoglu *et al.* (*25*). Briefly, 47.5 mL distilled water and 2.5 mL of 4 M HCl were added to 10 g of homogenized sample, which was then distilled using a distillation apparatus equipped with heating mantle (MS-EAM 9202-03; MTOPS, Yangju, Korea) at 100 °C for 10 min. After the transfer of 5 mL of distilled solution into the stoppered test tube, 5 mL thiobarbituric acid (TBA) reagent was added. Then, the solution was homogenized (Hg-15D; Daihan) and allowed to react with TBA for 35 min at 110 °C. At the end, absorbance was measured at 538 nm against a blank using a UV-Vis spectrophotometer (UV-1800; Shimadzu, Tokyo, Japan) and the results were expressed as mg malondialdehyde (MDA) per kg sample. The CIELab colour properties of meatballs were determined using a colorimeter (CR-400 Chroma Meter; Konica Minolta, Tokyo, Japan). Five consecutive measurements were taken from the surface of the samples with the illuminator D65 and an observer angle of 2°. The L*, a* and b* values were taken as the mean of five readings and were used to calculate total colour difference (∆*E*) according to the following equation (*26*):


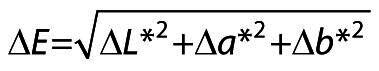
 /1/

All analyses were carried out on days 1, 3, 6 and 9 of the storage.

### Total phenolic content and antioxidant activity analysis

#### Extraction of phenolics

The phenolics were extracted by mixing 4 g of raw meatball sample with 16 mL of 80 % methanol, homogenization (Hg-15D; Daihan) at 2903×*g* for 1 min and centrifugation (Allegra X-22; Beckman Coulter, Brea, CA, USA) at 9299×*g* for 10 min (15 °C).

#### Total phenolic content

The slightly modified method of Nugboon and Intarapichet (*27*) was used to determine total phenolic content (TPC). Briefly, 200 μL of diluted extract, 1 mL of 0.2 M Folin-Ciocalteu reagent and 1 mL of sodium carbonate (75 g/L) solution were mixed in a glass test tube and allowed to rest for 3 min. Then, the solution was completed to 10 mL with distilled water and kept at room temperature in the dark for 90 min. After that, the absorbance of the solution was measured at 725 nm using a spectrophotometer (UV-1800; Shimadzu). The samples were analysed on days 1, 3, 6 and 9 of storage and the results were expressed as mg of gallic acid equivalents (GAE) per kg of sample.

#### DPPH˙ and ABTS^+^ radical scavenging assays

DPPH˙ radical scavenging activity of the meatballs was measured using the method proposed by Nugboon and Intarapichet (*27*) with slight modifications. For this purpose, 150 μL diluted extract were mixed with 2.85 mL of 0.1 mM DPPH˙ radical solution in pure methanol, which was then kept at room temperature in the dark for 30 min. The absorbance of the mixture was measured at 517 nm against pure methanol and the following equation was used to calculate the percentage of inhibition of the samples:


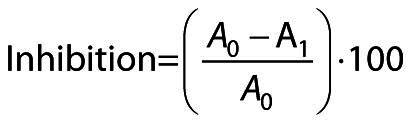
 /2/

where *A*_0_ is the absorbance of the blank solution and *A*_1_ is the absorbance of the sample solution.

The same procedures were used for the Trolox standard solution prepared at concentrations from 50 to 1000 μM, and the obtained percentage of inhibition values were plotted against the Trolox concentration to obtain the calibration curve (R^2^=0.9991). The results were then calculated using this calibration curve and considering all dilution factors, and were expressed as μmol of Trolox per kg of meatball sample. The analysis was carried out on days 1, 3, 6 and 9 of storage and each measurement was done in triplicate.

The method of Huang *et al.* (*28*), with slight modifications, was used to measure the ABTS^+^ radical scavenging activity of the samples. To prepare ABTS^+^ radical, 7 mM ABTS and 2.45 mM dipotassium peroxydisulfate solutions were mixed in equal volumes and the reagent was left in the dark at room temperature for 16 h. Then, the radical solution was diluted with pure methanol to an absorbance of 0.700±0.01 at 734 nm to obtain the working solution before the assay. For the spectrophotometric assay, 2 mL of ABTS^+^ radical working solution was added to 20 μL of aliquot and the measurements were taken at 734 nm against pure methanol after 7 min of reaction. The percentage inhibition values​​ of the samples were calculated using Eq. 2. The same procedures were used for Trolox standard solution prepared at concentrations from 50 to 2000 μM, and the obtained percentage of inhibition values ​​were plotted against the Trolox concentration to obtain the calibration curve (R^2^=0.9993). The results were then calculated using this calibration curve and considering all dilution factors, and were expressed as μmol of Trolox per kg of meatball sample. The analysis was carried out on days 1, 3, 6 and 9 of storage and each measurement was done in triplicate.

### Microbiological analysis

Microbiological analysis carried out as part of the study included the determination of *Enterobacteriaceae*, total yeasts and moulds (TYM), *Staphylococcus aureus* and total aerobic mesophilic bacteria (TAMB). Briefly, 10 g of raw sample was homogenized with 90 mL of 0.1 % peptone water using a Stomacher Lab-Blender 400 (Seward Medical, London, UK). Violet red bile dextrose agar was used to enumerate *Enterobacteriaceae* by pour plating method and the plates with samples were incubated at 37 °C for 24 h (*29*). Potato dextrose agar (5 days at (25±1) °C) was used for the count of TYM (*30*). Baird-Parker agar with egg yolk/tellurite emulsion was used for the determination of *S. aureus* and incubation was carried out at 37 °C for 30–48 h (*31*). The *S. aureus* colonies were verified using coagulase test. Plate count agar (PCA) was used for the TAMB and the plates were incubated at 30 °C for 24-48 h under aerobic conditions with pour plate method (*32*). All analyses were carried out on days 1, 3, 6 and 9 of storage and the results were expressed as log CFU/g.

### Sensory evaluation

Before the evaluation, each side of the meatball sample was cooked for 4 min at 180 °C on a preheated grill. After cooking, the samples were coded with letters and randomly presented to the panellists. Sensory parameters (preferences of colour, odour, taste, texture and overall acceptance) of cooked samples were evaluated by 30 untrained panellists (15 men and 15 women, aged 25-45). The panellists were chosen from people who habitually consume meatballs. All panellists were informed about the study at the beginning of testing. A 9-point hedonic scale was preferred for the analysis due to its widespread use. According to the scale, 1=dislike extremely, 2=dislike very much, 3=dislike moderately, 4=dislike slightly, 5=neither like nor dislike, 6=like slightly, 7=like moderately, 8=like very much and 9=like extremely for the relevant parameters. Water was consumed before the assessment of each sample (*33*). The sensory evaluations were carried out under the supervision and coordination of the project administrators. The informed permission was acquired from each individual before their involvement and appropriate protocols to protect the rights and privacy of all participants were used while conducting the study. The analysis was carried out on days 1, 3 and 6 of storage.

### Statistical analysis

The results were presented as mean value±standard error. The one-way analysis of variance (ANOVA) test was carried out using IBM Statistical Software (SPSS v. 22) (*34*). The Duncan’s multiple comparison test (with a 95 % confidence range) was used to identify significant differences between the results (p<0.05).

## RESULTS AND DISCUSSION

### Physicochemical properties

The physicochemical properties of meatballs produced within the scope of the study are shown in Table 1. As seen in the table, the increase in propolis amount did not significantly affect the pH values of the meatballs on the 1st and 3rd days of storage (p>0.05). However, this result changed on days 6 and 9, and significant differences between the samples were determined (p<0.05). It was observed that sample M1 had the lowest pH values (5.89 and 6.00) compared to the other samples on days 6 and 9. In addition, considering the entire storage period, the highest pH values were found on day 9 (p<0.05). According to the literature, the increase in the pH values during storage was also observed for other meat products such as burgers (*35*). It was reported that the increase in the pH values might be due to the production of endogenous or microbial enzymes such as protease and lipase triggered by bacterial growth during prolonged storage (*36*). According to the current results, the use of propolis suppressed the pH increase in the samples compared to the control, and it was concluded that especially 0.5 and 1 % propolis had a significant effect on pH control. Similar results were reported by Mehdizadeh and Langroodi (*37*), who investigated the effects of coating chicken breast meat with chitosan, propolis extract and *Zataria multiflora* Boiss oil on quality parameters and found that the combined treatment was more effective in controlling pH and microbial activity. Considering the *a*_w_ values, it was found that the addition of liquid propolis extract did not cause a difference between the samples (p>0.05). This was considered a positive result as the addition of propolis did not increase the moisture value of the samples (*38*). Meanwhile, although minor changes in *a*_w_ values ​​were observed depending on the storage period, this phenomenon did not lead to a significant result.

**Table 1:** The results of physicochemical analysis of raw meatballs stored at 4 °C

				Colour		
*t*(sample storage)/day	pH	*a*_w_	TBARS as MDA/(mg/kg)	L*	a*	b*
1						
Control	(5.91±0.07)^aEFGHI^	(0.95±0.00)^aB^	(2.50±0.07)^aCD^	(41.5±1.7)^aA^	(15.2±2.5)^aABC^	(12.2±0.4)^bDE^
M0.1	(5.91±0.04)^aEFGHI^	(0.95±0.00)^aB^	(2.6±0.1)^aC^	(43.3±2.6)^aA^	(15.6±2.0)^aAB^	(12.9±0.4)^abBCDE^
M0.3	(5.92±0.04)^aDEFGHI^	(0.95±0.01)^aB^	(2.2±0.1)^aDEF^	(42.9±1.7)^aA^	(17.9±1.2)^aA^	(13.2±0.2)^abABCDE^
M0.5	(5.90±0.05)^aFGHI^	(0.95±0.00)^aB^	(1.40±0.03)^bH^	(44.4±2.8)^aA^	(15.6±1.9)^aAB^	(14.0±1.6)^abAB^
M1	(5.84±0.04)^aI^	(0.95±0.00)^aB^	(1.5±0.2)^bGH^	(43.6±2.2)^aA^	(15.3±2.5)^aAB^	(14.5±1.3)^aA^
3						
Control	(5.98±0.00)^aDEF^	(0.96±0.00)^aA^	(3.22±0.08)^aB^	(41.0±2.0)^aAB^	(12.9±1.2)^aBCDE^	(12.2±0.2)^aDE^
M0.1	(5.94±0.09)^aDEFGH^	(0.96±0.00)^aA^	(3.15±0.02)^aB^	(42.6±0.8)^aAB^	(12.8±1.5)^aBCDE^	(12.6±0.4)^aBCDE^
M0.3	(5.94±0.03)^aDEFGH^	(0.96±0.00)^aA^	(2.7±0.2)^aC^	(44.1±3.0)^aAB^	(12.4±1.6)^aBCDE^	(13.0±1.2)^aABCDE^
M0.5	(5.91±0.07)^aEFGHI^	(0.95±0.00)^aB^	(1.87±0.06)^bEFG^	(43.9±3.9)^aAB^	(12.2±1.2)^aBCDE^	(12.9±1.3)^aBCDE^
M1	(5.87±0.04)^aHI^	(0.95±0.00)^aB^	(1.8±0.20)^bFG^	(43.6±0.7)^aAB^	(13.0±0.1)^aBCDE^	(13.7±1.7)^aABCD^
6						
Control	(5.99±0.03)^aCDE^	(0.96±0.00)^aA^	(3.4±0.4)^aB^	(42.0±4.0)^aAB^	(11.1±0.6)^aDE^	(12.1±0.2)^cE^
M0.1	(5.96±0.07)^abDEFG^	(0.95±0.00)^aB^	(3.2±0.1)^aB^	(43.7±1.7)^aAB^	(11.5±0.7)^aDE^	(12.9±0.4)^bBCDE^
M0.3	(5.95±0.06)^abDEFGH^	(0.96±0.00)^aA^	(2.7±0.2)^abC^	(44.5±2.3)^aAB^	(14.3±4.8)^aBCD^	(13.1±0.2)^bABCDE^
M0.5	(5.95±0.01)^abDEFGH^	(0.95±0.00)^aB^	(2.3±0.2)^bCDE^	(45.7±1.5)^aA^	(11.8±1.2)^aCDE^	(13.0±0.2)^bABCDE^
M1	(5.89±0.02)^bGHI^	(0.95±0.00)^aB^	(2.1±0.1)^bDEF^	(45.3±1.4)^aAB^	(11.2±1.7)^aDE^	(13.9±0.8)^aABC^
9						
Control	(6.17±0.02)^aA^	(0.95±0.00)^aB^	(3.9±0.3)^aA^	(40.8±2.8)^aB^	(13.2±0.6)^aBCDE^	(12.0±0.2)^cE^
M0.1	(6.15±0.02)^abA^	(0.95±0.00)^aB^	(3.5±0.2)^abB^	(43.7±2.5)^aAB^	(12.4±0.3)^abBCDE^	(12.2±0.3)^bcDE^
M0.3	(6.13±0.01)^bAB^	(0.96±0.01)^aA^	(3.0±0.1)^bcB^	(45.1±2.4)^aAB^	(14.2±2.0)^aBCD^	(12.4±0.4)^abcCDE^
M0.5	(6.06±0.00)^cBC^	(0.95±0.00)^aB^	(2.6±0.1)^cC^	(43.4±1.7)^aAB^	(11.3±0.6)^bDE^	(12.71±0.09)^abBCDE^
M1	(6.00±0.01)^dCD^	(0.95±0.00)^aB^	(2.5±0.2)^cCD^	(43.0±1.4)^aAB^	(10.7±0.2)^bE^	(13.0±0.5)^aABCDE^

Data represent mean value±standard deviation, *N*=3. Different lowercase letters in superscript within the same column indicate significant differences between the samples on the same day of storage for the same parameter (p<0.05). Different capital letters in superscript within the same column indicate significant differences between the samples during the entire storage period for the same parameter (p<0.05). M0.1-M1=meatball sample with the addition of 0.1, 0.3, 0.5 and 1 % of ethanolic extract of propolis, respectively, TBARS=2-thiobarbituric acid reactive substance, MDA=malondialdehyde

Lipid oxidation is the primary quality defect in meat and meat products that shortens the shelf life of the product. It is a chain reaction of free radicals consisting of three steps, namely initiation, propagation and termination (*39*). Peroxides, which are primary products of lipid oxidation, can undergo reactions that yield secondary oxidation products. Among them, MDA is one of the most important aldehydes as it gives rancid aromas to meat products even at low amounts. It is therefore used as a marker of lipid oxidation (*4*). In this context, it was observed that the TBARS values, expressed as MDA, of the meatball samples varied between 1.40 and 2.6 mg/kg sample on the first day and showed a significant increase up to 2.5-3.9 mg/kg sample on the 9th day. The addition of 0.5 and 1 % propolis to the meatball formulation led to a significant decrease in TBARS values (p<0.05), while lower amounts were not found to be effective (p>0.05). On the other hand, TBARS values increased as expected during storage and the highest value of 3.9 mg/kg was found in the control sample on the 9th day of storage (p<0.05). Although the perception of oxidized flavour depends on the experience and sensitivity of the individual, the threshold value of TBARS for a positive sensory perception of beef is reported to be 2 mg/kg (*40*). Since the TBARS values of control and M0.1 samples were higher than the threshold even at the beginning of storage, it could be concluded that lipid oxidation occurred during production, which could be due to prolonged exposure to oxygen. Although the TBARS values of samples M0.5 and M1 increased above the threshold value after 6 days of storage, they remained lower than of the other samples. Vargas-Sánchez *et al.* (*12*) found that lipid oxidation in beef patties started from the first day and increased continuously regardless of storage at low temperature (2 °C). The authors investigated the efficacy of ethanolic extract of propolis as an ingredient to inhibit lipid oxidation and confirmed that the addition of 2 % propolis extract into beef patties effectively delayed lipid oxidation after 4 and 8 days of storage at 2 °C compared to the control sample, depending on the polyphenol content of the extract. The results obtained in the current study also agree with those of Dos Reis *et al.* (*13*), who reported that the burger meat containing microencapsulated propolis co-product extract had lower TBARS values than the control and sodium erythorbate-treated samples during storage at -15 °C. It can be concluded that the addition of propolis at 0.5 and 1 % was an effective way to delay lipid oxidation in meatballs stored under refrigerated conditions.

Colour and colour stability are the most important parameters for the quality and freshness of meat; however, changes in the colour during storage affect consumer preference (*41*). In the current study, the addition of propolis to the meatball formulation did not significantly change the L* and a* values of the samples on the first day (p>0.05), while it caused a linear increase in the b* values of the samples with added propolis (12.9-14.5) compared to the control sample (12.2) (p<0.05). On the other hand, although there was no significant change in the L* value of the samples depending on the storage time, the a* values, which varied between 15.2 and 17.9 on the first day, decreased to a range of 10.7-14.2 on the 9th day. Interestingly, the lowest a* values were recorded on the last day of storage at 11.3 and 10.7 in samples M0.5 and M1, respectively (p<0.05). The b* values of the samples also changed depending on storage time, from 12.2-14.5 on the first day to 12.0-13.0 on the last day, and the highest b* values were found in the samples with added propolis throughout the storage period (p<0.05). It is known that long-term storage in an aerobic environment causes the transformation of oxymyoglobin (bright red colour) into metmyoglobin (brown colour) and this change makes meat and meat products unacceptable (*12*). In this context, in view of the current results, changes in the a* value can be prevented in further studies by using vacuum packaging and modified atmosphere applications that prevent the contact of the samples with oxygen. In a previous study, Vargas-Sánchez *et al.* (*5*) found that the addition of 2 % ethanolic propolis to beef and pork patties did not affect the L* values ​​of the samples regarding the initial and storage time. On the other hand, it was observed that the initial a* and b* values ​​of all samples decreased significantly depending on the storage at 2 °C in the dark for 9 days, but this decrease was delayed in the samples with added propolis compared to the control sample. In another study, it was found that increasing the amount of propolis extract (0.25, 0.50, 1 and 3 %) obtained after 15 days using 70 % ethanol did not affect the L*, a* and b* values ​​of fresh trout fillets (*42*). According to the study by El Sheikha *et al.* (*43*), the L*, a* and b* values ​​of chicken breast meat samples coated with carboxymethyl cellulose (CMC) containing different amounts of ethanolic propolis extract (1, 2, 3 and 4 %) did not change initially. However, a significant increase was observed in the L* and a* values ​​of the sample coated with CMC containing 4 % propolis compared to the other samples depending on the storage time of 16 days at 2 °C.

### The results for total phenolic content and antioxidant activity

Reformulation studies using natural ingredients, extracts or compounds with high antioxidant capacity such as propolis are of great importance for both the meat industry and consumer perception (*44*, *45*). In this regard, the results of the determination of total phenolic content (TPC) and antioxidant activity values (DPPH˙ and ABTS^+^ radical scavenging assays) of raw meatball samples during storage are shown in Table 2. According to the results, the addition of propolis as a natural antioxidant compound to the formulation gradually increased the TPC of meatballs, and this increase became significant at amounts higher than 0.3 % compared to the control sample. On the other hand, considering the changes in the TPC values of meatballs during storage, a similar decreasing trend was observed in each sample, as expected, and the lowest values were obtained on day 9 of storage compared to the initial results. In a previous study, beef and pork patties treated with propolis extract showed higher TPC values than the control sample throughout storage (*5*), which are consistent with the current results. Similar to the TPC results, the DPPH˙ and ABTS^+^ radical scavenging values were also significantly increased with the addition of propolis. In another study, the addition of 5 % spray-dried propolis to an optimised fish burger formulation with 10 % potato flakes and 9 % extra virgin olive oil resulted in about three times higher phenolic content and about four times higher DPPH˙ radical scavenging activity than in the control (*46*). Meanwhile, both the DPPH˙ and ABTS^+^ radical scavenging values showed similar trends, with values gradually decreasing during storage and being the lowest at the end of storage. The antioxidant activity of propolis can be attributed to its high phenolic content since these compounds contribute directly to the antioxidant activity by acting as reducing agents, hydrogen donors and singlet oxygen quenchers (*47*). The propolis has been reported to contain mainly flavonoid aglycones, which prevent lipid oxidation by breaking free radical reactions (*48*). Therefore, it was reasonable to associate the decrease in TBARS values (Table 1) with the increased DPPH˙ and ABTS^+^ radical scavenging activity of meatballs with propolis, especially at 0.5 and 1 %.

**Table 2:** Total phenolic content and antioxidant activity of raw meatball samples stored at 4 °C

*t*(sample storage)/day	Total phenolic content as GAE/(mg/kg)	DPPH as Trolox/(µmol/kg)	ABTS as Trolox/(µmol/kg)
1			
Control	(630±15)^cDE^	(116.3±7.1)^dHI^	(500±17)^eF^
M0.1	(650±9)^cD^	(157.2±9.3)^cG^	(573±12)^dD^
M0.3	(680±11)^bcC^	(314±9)^bD^	(659±21)^cC^
M0.5	(692±11)^bC^	(506±12)^aB^	(729±19)^bB^
M1	(789±15)^aA^	(532±13)^aA^	(850±17)^aA^
3			
Control	(615±18)^cE^	(115±11)^dHI^	(395±8)^dI^
M0.1	(628±11)^cDE^	(134±10)^dHI^	(380±11)^dIJ^
M0.3	(637±6)^cDE^	(250±9)^cE^	(453±17)^cGH^
M0.5	(682±11)^bC^	(405±20)^bC^	(514±19)^bEF^
M1	(757±12)^aB^	(450±15)^aB^	(579±13)^aD^
6			
Control	(396±6)^dJ^	(91.1±14.0)^cJ^	(327±6)^eK^
M0.1	(455±16)^cdHI^	(126.4±14.5)^bcHI^	(367±8)^dJ^
M0.3	(469±14)^cdGH^	(131.5±12.1)^bcC^	(434±14)^cH^
M0.5	(538±20)^bF^	(159.0±7.2)^bG^	(460±12)^bG^
M1	(683±26)^aC^	(236±10)^aE^	(529±18)^aE^
9			
Control	(388±11)^dJ^	(73.9±14.3)^cJ^	(240.3±5.6)^eM^
M0.1	(408±18)^cdJ^	(111.4±7.2)^bcI^	(281±11)^dL^
M0.3	(435±20)^cI^	(115.8±11.2)^bcHI^	(399±12)^cI^
M0.5	(492±12)^bG^	(126.4±7.1)^bHI^	(444±14)^bGH^
M1	(640±12)^aDE^	(197.2±14.5)^aF^	(495±10)^aF^

Data represent mean value±standard deviation, *N*=3. Different lowercase letters in superscript within the same column indicate significant differences between the samples on the same day of storage for the same parameter (p<0.05). Different capital letters in superscript within the same column indicate significant differences between the samples during the entire storage period for the same parameter (p<0.05). M0.1-M1=meatball sample with the addition of 0.1, 0.3, 0.5 and 1 % of ethanolic extract of propolis, respectively

### Results of microbiological analyses

In the present study, the total aerobic mesophilic bacteria (TAMB), *Enterobacteriaceae*, total yeasts and moulds (TYM) and *Staphylococcus aureus* counts were determined in order to investigate the effects of different amounts of propolis on the hygiene indicators in the meatballs. The results are shown in Table 3. According to the results, the propolis was effective in decreasing the microbiological counts. Although there was an increase in bacterial counts towards the end of the storage, it was determined that this increase was suppressed in the meatballs with added propolis compared to the control sample. Considering the TAMB counts, the control sample was found to be significantly different from the other samples enriched with propolis, except for sample M0.1 on days 3 and 9 of storage (p<0.05). On the other hand, an increase in TAMB counts was observed depending on the storage time, but this increase was delayed with the addition of propolis. In particular, when the amount of propolis added to the meatballs exceeded 0.3 %, significantly lower TAMB counts were found throughout the storage (p<0.05). In this regard, sample M1 had significantly lower TAMB count than the other samples at all storage times (p<0.05). Similarly, Kim *et al.* (*49*) reported that the addition of 1 % propolis extract to the meatball formulation clearly inhibits the TAMB growth. In contrast to TAMB counts, the addition of propolis did not affect the counts of *Enterobacteriaceae* on day 1 of storage (p>0.05). Although there was a slight increase in the counts after the 3rd day of storage, *Enterobacteriaceae* counts were determined to be significantly lower, especially in samples containing 0.3, 0.5 and 1 % propolis, than in the control sample (p<0.05). In a study conducted by Gedikoğlu (*50*), commercial water extract of propolis was added to the raw beef meatballs and the samples were stored at 4 °C for 7 days. According to the research, the addition of propolis decreased the *Enterobacteriaceae* counts by 2.24 log CFU/g (31.9 %) and TAMB counts by 2.42 log CFU/g (24.9 %). The addition of propolis at amounts higher than 0.3 % led to a dramatic decrease in the TYM counts at day 1 of storage (p<0.05). Although almost similar values were found on days 3 and 6 of storage between the control and meatballs containing propolis (p>0.05), the M0.5 and M1 samples showed significantly lower TYM counts at the end of the storage, regardless of the total increase (p<0.05). Similarly, Ali *et al.* (*51*) found that the addition of 0.6 % propolis to sausages improved shelf life by more than one week by reducing the proteolytic, lipolytic and TYM counts. Regarding the *S. aureus* counts, an insignificant decrease was observed at amounts up to 0.5 % on the first day of storage (p>0.05), while it became significant in samples M0.5 and M1 (p<0.05). Moreover, samples M0.5 and M1 had significantly lower counts of *S. aureus* than the control sample, regardless of the total increase throughout the storage (p<0.05). These results are consistent with the findings from the literature, where the effective amount of propolis is reported to be in the range of 0.5-2 % (*49*, *51*, *52*). Based on the results of microbiological analysis, it could therefore be concluded that the amount of propolis added to the meatballs should not be less than 0.5 % under these conditions.

**Table 3:** Microbiological analysis of raw meatball samples stored at 4 °C

	*N*/(logCFU/g)			
*t*(sample storage)/day	TAMB	*Enterobacteriaceae*	TYM	*S. aureus*
1				
Control	(5.82±0.07)^aJ^	(4.33±0.09)^aHI^	(3.5±0.2)^aG^	(3.8±0.3)^aIJ^
M0.1	(5.64±0.01)^bK^	(4.2±0.1)^aIJ^	(3.2±0.3)^abHI^	(3.6±0.3)^abJK^
M0.3	(5.47±0.06)^cL^	(4.2±0.1)^aJ^	(3.0±0.1)^bcIJ^	(3.5±0.3)^abK^
M0.5	(5.39±0.05)^cL^	(4.2±0.1)^aIJ^	(2.92±0.07)^bcJ^	(3.2±0.2)^bcL^
M1	(5.13±0.02)^dM^	(4.16±0.03)^aJ^	(2.8±0.2)^cJ^	(2.79±0.09)^cM^
3				
Control	(6.21±0.07)^aI^	(4.49±0.01)^aGH^	(3.9±0.1)^aF^	(4.4±0.2)^aG^
M0.1	(6.2±0.1)^aI^	(4.45±0.02)^aGH^	(3.86±0.02)^aF^	(4.19±0.07)^bGH^
M0.3	(5.88±0.08)^bJ^	(4.34±0.00)^bHI^	(3.81±0.04)^aF^	(4.13±0.05)^bH^
M0.5	(5.54±0.09)^cKL^	(4.20±0.01)^cIJ^	(3.4±0.2)^bGH^	(4.07±0.07)^bHI^
M1	(5.2±0.1)^dM^	(3.82±0.08)^dK^	(3.3±0.2)^bGH^	(3.85±0.08)^cIJ^
6				
Control	(7.2±0.1)^aC^	(5.3±0.1)^aD^	(5.4±0.2)^aE^	(5.50±0.07)^aDE^
M0.1	(7.0±0.1)^bDE^	(5.01±0.03)^bE^	(5.48±0.02)^aE^	(5.26±0.20)^bE^
M0.3	(6.65±0.03)^cGH^	(4.87±0.06)^bEF^	(5.45±0.03)^aE^	(4.97±0.03)^cF^
M0.5	(6.5±0.1)^cH^	(4.56±0.09)^cG^	(5.24±0.16)^aE^	(4.72±0.09)^dF^
M1	(6.14±0.06)^dI^	(4.22±0.10)^dIJ^	(5.30±0.27)^aE^	(4.41±0.07)^eG^
9				
Control	(7.81±0.05)^aA^	(6.66±0.08)^aA^	(6.8±0.1)^aA^	(6.2±0.2)^aA^
M0.1	(7.6±0.2)^aB^	(6.5±0.2)^aB^	(6.7±0.2)^abAB^	(6.0±0.1)^abAB^
M0.3	(7.1±0.2)^bCD^	(5.68±0.07)^bC^	(6.5±0.1)^bcBC^	(5.82±0.08)^bBC^
M0.5	(6.86±0.05)^bcEF^	(5.22±0.08)^cD^	(6.27±0.07)^cC^	(5.57±0.08)^cCD^
M1	(6.8±0.1)^cFG^	(4.8±0.1)^dF^	(5.95±0.07)^dD^	(5.35±0.05)^dDE^

Data represent mean value±standard deviation, *N*=3. Different lowercase letters in superscript within the same column indicate significant differences between the samples on the same day of storage for the same parameter (p<0.05). Different capital letters in superscript within the same column indicate significant differences between the samples during the entire storage period for the same parameter (p<0.05). M0.1-M1=meatball sample with the addition of 0.1, 0.3, 0.5 and 1 % of ethanolic extract of propolis, respectively, TAMB=total aerobic mesophilic bacteria, TYM=total yeast and mould

### Sensory evaluation results

The results of the sensory evaluation of the cooked meatball samples are shown in Table 4. As part of the study, the sensory evaluation was conducted on samples stored for 6 days. The 9th day was not included in the evaluation as the chemical and microbiological results of the samples were considered. According to the results, it was found that the amount of propolis and the storage time did not cause a significant difference in the colour liking scores of the cooked samples, which was between 8.2 and 8.6 throughout the storage. The addition of propolis also did not cause a significant difference in the evaluation of odour, taste and overall acceptance parameters compared to the control sample. However, the highest scores were obtained for all samples on the first day, which then they decreased with increasing storage time in all samples (p<0.05). For instance, odour and taste scores on the first day varied between 8.1 and 8.6, while on the 6th day of storage the scores decreased to 6.8-7.4 and 6.0-6.4, respectively. Although the addition of propolis slightly increased the texture scores compared to the control sample, the difference was not significant. According to the results for overall acceptability, all samples received high scores between 8.0 and 8.8 on the first day. Only the sample enriched with 1 % propolis affected the overall acceptability of the panellists during the entire storage time. This can undoubtedly be attributed to the intense aroma of the propolis.

**Table 4:** Sensory evaluation of cooked meatball samples

*t*(sample storage)/day	Colour liking	Odour	Taste	Texture	Overall acceptance
1					
Control	(8.4±0.2)^aA^	(8.6±0.2)^aA^	(8.5±0.3)^aA^	(8.6±0.4)^aA^	(8.4±0.3)^aAB^
M0.1	(8.5±0.4)^aA^	(8.4±0.3)^aAB^	(8.4±0.5)^aA^	(8.5±0.4)^aA^	(8.8±0.2)^aA^
M0.3	(8.5±0.4)^aA^	(8.4±0.5)^aABC^	(8.6±0.3)^aA^	(8.5±0.4)^aA^	(8.7±0.2)^aA^
M0.5	(8.6±0.3)^aA^	(8.2±0.2)^aABC^	(8.5±0.4)^aA^	(9.00±0.00)^aA^	(8.5±0.1)^aAB^
M1	(8.6±0.2)^aA^	(8.1±0.6)^aABC^	(8.1±0.4)^aAB^	(9.00±0.00)^aA^	(8.0±0.3)^abBC^
3					
Control	(8.4±0.4)^aA^	(7.8±0.1)^aABCD^	(7.6±0.4)^aBC^	(8.5±0.2)^aA^	(7.7±0.4)^bcCD^
M0.1	(8.4±0.2)^aA^	(7.8±0.4)^aBCD^	(7.7±0.5)^aBC^	(8.6±0.4)^aA^	(7.6±0.6)^bcCD^
M0.3	(8.5±0.2)^aA^	(7.8±0.4)^aABCD^	(7.6±0.2)^aBC^	(8.5±0.3)^aA^	(7.3±0.5)^abCDE^
M0.5	(8.5±0.4)^aA^	(7.8±0.4)^aABCD^	(7.4±0.6)^aBC^	(8.8±0.2)^aA^	(7.2±0.72^aDE^
M1	(8.6±0.4)^aA^	(7.6±0.2)^aCDE^	(7.2±0.4)^aC^	(9.00±0.00)^aA^	(6.8±0.4)^cEF^
6					
Control	(8.2±0.6)^aA^	(6.8±0.5)^aF^	(6.3±0.2)^aD^	(8.5±0.4)^aA^	(6.5±0.2)^aFG^
M0.1	(8.2±0.3)^aA^	(6.9±0.4)^abEF^	(6.4±0.3)^aD^	(8.5±0.5)^aA^	(6.4±0.1)^abFG^
M0.3	(8.4±0.4)^aA^	(7.2±0.6)^aDEF^	(6.4±0.5)^aD^	(8.4±0.3)^aA^	(6.2±037)^abFG^
M0.5	(8.4±0.3)^aA^	(7.4±0.3)^aDEF^	(6.2±0.2)^aD^	(8.5±0.4)^aA^	(6.3±0.4)^abFG^
M1	(8.5±0.2)^aA^	(7.0±0.6)^aEF^	(6.0±0.4)^aD^	(8.6±0.2)^aA^	(5.9±0.2)^bG^

Data represent mean value±standard deviation, *N*=3. Different lowercase letters in superscript within the same column indicate significant differences between the samples on the same day of storage for the same parameter (p<0.05). Different capital letters in superscript within the same column indicate significant differences between the samples during the entire storage period for the same parameter (p<0.05). M0.1-M1=meatball sample with the addition of 0.1, 0.3, 0.5 and 1 % of ethanolic extract of propolis, respectively

Considering the studies using propolis extract in various meat and meat products, Payandan *et al.* (*53*) investigated the effectiveness of both ethanolic and aqueous extracts of propolis added to minced carp meat at 3, 5 and 7 % on microbiological and sensory parameters during 9 days of storage at 4 °C and found that there was no difference in colour and texture values ​​of all samples on the first day. Interestingly, the samples enriched with the highest amount of propolis obtained with both solvents appeared to have higher initial odour and overall acceptability scores than the control sample. On the other hand, sensory scores of all samples decreased depending on the storage time, and it was found that the scores on the 6th day were between 4.5 and 6.5 on a 10-point scale. In addition, all samples had the lowest scores on day 9, but this decrease was particularly suppressed in the samples with ethanolic extract of propolis compared to the control sample. In another study, Fadhil (*54*) investigated the effects of the addition of aqueous propolis extract in different ratios on the shelf life and various quality parameters of chicken meat and reported that propolis addition (5-15 %) positively affected the odour parameter. In addition, the overall' acceptability of the samples enriched with 5 and 10 % aqueous propolis was comparable to that of the control sample, and the author concluded that the aqueous extract of propolis had a potential application as a natural preservative in chicken meat. Considering that ethanol is able to denature proteins by disrupting the non-covalent bonds in the tertiary structures (*55*), the use of ethanol at lower volume fractions in the extract solution (<70 %) or the use of other solvents such as water in propolis extraction can be tried in future studies to delay the formation of off-odours that may result from this phenomenon. There are also studies in the literature that use different “forms” of propolis. For instance, Bernardi *et al.* (*56*) used both free and microencapsulated propolis in an Italian-type salami product and found that the samples with added propolis generally had the most acceptable appearance during the 90-day storage time. However, the same study found that there were relative differences in aroma and overall acceptability criteria for the propolis-enriched salami samples compared to the control sample due to the persistent aroma and taste of propolis. In the study conducted by Dos Reis *et al.* (*13*) on the effect of microencapsulated propolis (0.3 g/kg) on ​​the stability of burger meat during storage at -15 °C, it was determined that the colour, appearance and texture properties of the burger meat with added propolis were at an ideal level, while the aroma and flavour were lower than of the control sample. In that study, the burger meat samples with sodium erythorbate and propolis had an acceptance rate of 72.52 and 63.80 %, respectively. Based on these results, the encapsulation of propolis using different techniques and wall materials such as gelatine, maltodextrin, starch and chitosan could help to change the perception of its intense aroma on the organoleptic properties of the samples.

## CONCLUSIONS

In the present study, different amounts of propolis (0.1, 0.3, 0.5 and 1 %) were added to the meatballs to improve the oxidative and microbiological quality, and thus extend the storage time of the raw samples at 4 °C. The results showed that the addition of propolis did not significantly affect the pH values ​​of the meatball samples on the first day, while it suppressed the increase in pH values ​​depending on the storage time. The addition of liquid propolis at all amounts did not have an effect on the *a*_w_ values of the samples. TBARS values increased with storage time in all samples, but it was observed that the lipid oxidation phenomenon was delayed in the samples with the addition of propolis. The propolis in the meatball formulation did not significantly change the L* and a* values, but it increased the b* values of the samples on the first day. During storage, no significant change was observed in the L* values of the samples, while there was a more significant decrease in the a* values ​​than in the b* parameter of the samples. Moreover, the total phenolic content, DPPH˙ and ABTS^+^ radical scavenging values increased significantly with increasing propolis amount. It was reasonable to associate the decreased TBARS values with the increased antioxidant activity of the meatballs containing propolis. The propolis was effective in delaying all microbiological criteria tested, but if a longer storage time is desired, the amount should not be below 0.5 %. According to the sensory results, it should be considered that increasing the amount of propolis may affect the overall acceptability. In conclusion, certain amounts of propolis can be used as a natural antioxidant and antimicrobial ingredient in meatballs stored at 4 °C to improve the oxidative and microbiological properties of the product.
